# Improved antibody breadth with an extended primary dose interval of COVID-19 vaccine is overcome by boosters

**DOI:** 10.3389/fimmu.2025.1529134

**Published:** 2025-03-27

**Authors:** Jessica I. Ahmed, Samantha J. Krosta, Mandy N. Reimer, Winnie Cheung, Christine Mesa, Carmen Lopez, Rayeil J. Chua, Farah Alsattari, Alyssia Robinson, Kathy Manguiat, Naima Jahan, Bernard Abrenica, Angela Harris, Karla Cachero, Rissa Fabia, Jonathan Walker, Myo Minn Oo, Derek Stein, Hezhao Ji, Ruey-Chyi Su, Paul J. McLaren, Lyle R. McKinnon, T Blake Ball, Heidi Wood, John Kim, Sandra A. Kiazyk, Catherine M. Card

**Affiliations:** ^1^ Division of Sexually-Transmitted and Blood-Borne Infections, Public Health Agency of Canada, Winnipeg, MB, Canada; ^2^ Division of Mycobacteriology, Vector-borne and Prion Diseases, Public Health Agency of Canada, Winnipeg, MB, Canada; ^3^ Department of Medical Microbiology and Infectious Diseases, University of Manitoba, Winnipeg, MB, Canada; ^4^ Department of Serology and Parasitology, Cadham Provincial Laboratory, Winnipeg, MB, Canada; ^5^ Department of Serology and Parasitology, Centre for the AIDS Programme of Research in South Africa (CAPRISA), Durban, South Africa

**Keywords:** COVID-19, mRNA vaccine, dose interval, antibody, breadth, neutralization, T cell, booster

## Abstract

**Introduction:**

During rollout of mRNA-based COVID-19 vaccines, several jurisdictions extended the interval between the first and second doses to prioritize wider population access to limited vaccine supply. This study evaluated the effects of an extended dose interval on development of antibody and cell-mediated responses following the primary dose series and a subsequent booster dose.

**Methods:**

Blood samples were collected from mRNA COVID-19 vaccine recipients at baseline and longitudinally after each dose. Samples were analyzed for SARS-CoV-2-specific antibody titers, neutralizing antibodies and memory T cell responses.

**Results:**

An extended dose interval was associated with improved breadth of neutralizing antibody responses against both ancestral and early SARS-CoV-2 variants, but not Omicron variants. Dose interval had no impact on the development of antigen-specific memory T cell responses, the memory or T helper phenotypes of responding T cells or cytokine production. The effects of the primary dose interval on immune outcomes were no longer evident after a third dose of mRNA vaccine.

**Discussion:**

An extended primary dose interval resulted in short-term benefits to humoral immunity but these were transient in the context of subsequent exposures. However, in addition to the public health benefits of wider population access to vaccines, the short-term immunological benefits of extending the dose interval may have been sustained in the absence of boosters. These findings underscore the importance of evaluating dosing intervals during the development of future vaccine candidates.

## Introduction

The coronavirus disease 2019 (COVID-19) pandemic led to the rapid development of mRNA-based COVID-19 vaccines following the identification and sequencing of severe acute respiratory syndrome coronavirus 2 (SARS-CoV-2). Due to high demand and limited supply, a phased approach was applied to the rollout of COVID-19 vaccines, in which priority access to vaccination was granted to frontline workers and people at high risk of severe disease. However, faced with vaccine shortages, Canada’s National Advisory Committee on Immunization recommended a dose-sparing strategy whereby second doses would be postponed for up to 4 months in favor of offering wider population access to the first dose (1).

Clinical trials initially demonstrated high efficacy of the Pfizer-BioNTech BNT162b2 and Moderna mRNA1273 vaccine formulations with a 3- or 4-week dose interval, respectively (2, 3). Nevertheless, a single vaccine dose showed excellent protection against ancestral SARS-CoV-2, and modeling studies suggested that delaying the second dose to favor broader first dose coverage would avert infections, hospitalizations, and deaths (4–6). This was supported by clinical trials of the AstraZeneca COVID-19 vaccine, which demonstrated superior efficacy of the two-dose regimen with longer intervals between doses (7).

Emerging data from populations that implemented the delayed second dose have since demonstrated that an extended dose interval led to higher antibody titers (8–14) with improved viral neutralization (8–10, 12). Overall, lengthening the dose interval did not appear to impact the development of antigen-specific T cells (9–11, 15). These observations are consistent with the observed enhancement of extended prime-boost dose intervals on antibody, but not T-cell responses in vaccinated mice (16). Despite these encouraging observations, questions remain regarding the impact of dose interval on protective immunity, particularly in the context of emerging variants and booster shots.

The present study enrolled vaccine recipients with either a standard (3-week) or extended (6–12-week) interval between the first two doses of the BNT162b2 vaccine. A comprehensive longitudinal analysis was conducted to assess the impact of dose interval on immune effector mechanisms including antibody titers, neutralizing antibodies, and the magnitude and quality of memory T-cell responses. We show here that a delayed second dose is associated with sustained antibody titers and improved breadth of neutralizing antibodies against SARS-CoV-2 variants that are closely related to the ancestral strain, but not more distantly related Omicron sublineages. The dose interval had no impact on the magnitude of memory T-cell development or the quality of T-cell responses. Importantly, the effects of the dose interval were transient, as all immune outcomes were similar between groups after a third (booster) dose of an mRNA-based COVID-19 vaccine.

## Methods

### Study participants

The study participants were laboratory workers from two public health labs in Manitoba, Canada, who were offered priority access to COVID-19 vaccines. The inclusion criteria for this analysis were adults aged 18–65 in generally good health who were scheduled to receive an mRNA COVID-19 vaccine. The exclusion criteria included prior history of COVID-19 infection (self-reported and/or identified by the presence of anti-nucleocapsid antibodies) and history of autoimmune disease or current use of immunomodulatory medication. A total of 70 individuals (*n* = 41 for the standard interval group and *n* = 29 for the extended interval group) met the criteria for inclusion in this analysis. The study participants provided biological samples and responded to demographic and basic clinical questionnaires.

### Sample collection and processing

Blood samples were collected prior to vaccination (baseline) then longitudinally after each vaccine dose. Venous blood was collected into EDTA tubes by standard venipuncture phlebotomy. Blood tubes were centrifuged for 7 min at 450×*g* to separate plasma, which was stored at −80°C. Peripheral blood mononuclear cells (PBMCs) were isolated from plasma-depleted blood using Lymphoprep in SepMate-50 tubes (STEMCELL Technologies, Vancouver, BC, Canada) according to the manufacturer’s instructions. PBMCs were counted using a Countess 3 FL automated cell counter (Thermo Fisher Scientific, Waltham, MA, USA) then cryopreserved in RPMI 1640 medium (Life Technologies, Carlsbad, CA, USA) supplemented with 40% heat-inactivated fetal bovine serum (FBS; GIBCO, Grand Island, NY, USA) and 15% dimethylsulfoxide (DMSO: MilliporeSigma Canada Ltd., Oakville, ON, Canada).

### Serology

Serological analysis of plasma anti-Spike (S1) IgG, anti-receptor-binding domain (RBD) IgG, and anti-nucleocapsid (NC) IgG was performed using the BioPlex 2200 SARS-CoV-2 IgG assay (Bio-Rad, Mississauga, ON, Canada). Plasma samples were thawed and vortexed after 30 min and then centrifuged at 2,000×*g* for 1 min. Each sample was queued for the BioPlex 2200 SARS-CoV-2 IgG assay undiluted and diluted on-board at a 1 in 32 dilution. A sample with a result >8,000 BAU/mL for any of the targets was manually diluted to achieve a quantitative result within the reportable range. Antibodies were quantified using the World Health Organization (WHO) International Immunoglobulin Standard for anti-SARS-CoV-2 (17) (NIBSC code 20/136, National Institute for Biological Standards and Control, Potters Bar, Hertfordshire, England). The presence of any SARS-CoV-2-specific antibodies in baseline samples or anti-NC antibodies at any time point was interpreted as evidence of prior infection and those individuals were excluded from the analysis.

### Viral stock production

Vero E6 cells (ATCC, Manassas, VA, USA) were cultured in Dulbecco’s modified Eagle’s medium (DMEM), high glucose with L-glutamine (HyClone, San Angelo, TX, USA) supplemented with 10% FBS (GIBCO, Grand Island, NY, USA). Ancestral (hCoV-19/Canada/ON-ON-VIDO-01-2/2020, EPI_ISL_425177) or Delta (B.1.167.2) SARS-CoV-2 stocks were created by infection of Vero E6 cells at a multiplicity of infection (MOI) of 0.1. After cell adsorption for 1 h at 37°C and 5% CO_2_, DMEM supplemented with 2% FBS was added before incubation for 1 h at 37°C. Light microscopy was used daily to detect cytopathic effects (CPEs) in infected cells. The virus supernatant collected 72 h post-infection was centrifuged at 525×*g* for 10 min at 4°C and then aliquoted and stored at −80°C for future use. SARS-CoV-2 stocks were titrated according to a previously described plaque assay (18).

### Plaque reduction neutralization test

Quantification of the levels of neutralizing antibodies against SARS-CoV-2 was done using the plaque reduction neutralization test (PRNT), as previously described (19). This method was validated according to the guidelines outlined by the WHO and was the reference standard for the quantification of neutralization. Plasma samples were diluted at 1:10 in DMEM supplemented with 2% FBS and then heat-inactivated at 56°C for 30 min before diluting further in a two-fold dilution series from 1:10 to 1:640. In a 96-well plate, 100 plaque-forming units (PFUs) of SARS-CoV-2 in 100 µL was mixed at a 1:1 volume with the sample, yielding a final virus concentration of 50 PFU/100 µL. No neutralization, 50% neutralization, and 90% neutralization controls were prepared by diluting 50 PFU/100 µL, 25 PFU/100 µL, and 5 PFU/100 µL, respectively, along with a no-virus control. Plates were incubated for 1 h at 37°C and then plated in duplicate on 12-well plates with confluent Vero E6 cells. Plates were incubated for 1 h at 37°C for adsorption and then monolayers were overlaid with 1.5 mL of prepared modified Eagle’s medium (Temin’s modification), no phenol red with L-glutamine, 8% FBS, 2% non-essential amino acids solution (GIBCO, Grand Island, NY, USA), and 1.5% sodium bicarbonate (GIBCO, Grand Island, NY, USA) supplemented with 3% (w/v) carboxymethylcellulose (MilliporeSigma Canada Ltd., Oakville, ON, Canada). After a 3-day incubation at 37°C, the liquid overlay was removed and cells were fixed with 10% neutral buffered formalin solution (MilliporeSigma Canada Ltd., Oakville, ON, Canada) for 1 h at room temperature. Monolayers were stained with 0.5% (w/v) crystal violet (MilliporeSigma Canada Ltd., Oakville, ON, Canada) in 20% ethanol for 10 min and then washed with 20% ethanol and air dried. For each dilution, the average number of plaques was calculated and compared with the average number of plaques for 50% neutralization and 90% neutralization controls. The neutralizing antibody titer in PRNT assays is reported as the highest reciprocal plasma dilution at which 50% or 90% reduction in plaque formation is achieved for PRNT50 and PRNT90, respectively. Reciprocal endpoint plasma dilutions include <20 (negative), 20, 40, 80, 160, 320, or ≤640. For statistical analysis purposes, serological samples with a PRNT50 or PRNT90 reciprocal titer of <20 were reported as 1.

### Angiotensin-converting enzyme 2 binding inhibition assay

Plasma samples were thawed, centrifuged for 3 min at 2,000×*g* at 4°C, and then diluted 1:100 in Diluent 100 buffer (Meso Scale Discovery, Rockville, MD, USA). The V-PLEX SARS-CoV-2 Key Variant RBD Panel 1 [angiotensin-converting enzyme 2 (ACE2)] Kit (Meso Scale Discovery, Rockville, MD, USA) was used to quantify antibodies that block the binding of ACE2 to SARS-CoV-2 RBD antigens derived from Ancestral, B.1.1.7 (Alpha), B.1.351 (Beta), B.1.617.2 (Delta), and Omicron variants BA.1, BA.2, BA.2.12.1, BA.2.75, and BA.4/BA.5. The assay was run according to the manufacturer’s instructions and plates were read on a 1300 Meso QuickPlex SQ 120MM (Meso Scale Discovery, Rockville, MD, USA). An in-house positive internal control was prepared by identifying a plasma sample with high neutralizing capability against all variants included in the panel and run on every plate. The negative control was Diluent 100.

Data analysis was done using the Discovery Bench 4.0 software (Meso Scale Discovery, Rockville, MD, USA). Results are reported as percent (%) inhibition of ACE2 binding by the sample, with the negative control serving as a 0% inhibition reference. Baseline plasma samples were used to determine the background level of non-specific inhibition. This threshold was calculated as the mean + 2 standard deviations of all baseline samples across all tested variants. Based on this analysis, samples with % inhibition below 40% were considered to be below the cutoff and were assigned 0% inhibition for subsequent analyses.

### T-cell activation-induced marker assay

Cryopreserved PBMCs were thawed and rested in RPMI 1640 media (Life Technologies) supplemented with 10% FBS (GIBCO, Grand Island, NY, USA) and 1% penicillin/streptomycin (Thermo Fisher Scientific, Waltham, MA, USA) herein referred to as R10, for 4 h at 37°C. For stimulation, 1 × 10^6^ PBMCs were cultured in 200 µL per well in 96-well round bottom plates or as low as 5 × 10^5^ in cases of low sample availability. Cells were stimulated for 16 h with a pool of overlapping SARS-CoV-2 Spike peptides or CEFX, a control peptide pool derived from CMV, EBV, influenza, and other pathogens, at 1 µg/peptide/mL (all peptides from JPT Innovative Peptide Technologies, Berlin, Germany). The positive control stimulation used was Dynabeads Human T-Activator CD3/CD28 (Thermo Fisher Scientific, Waltham, MA, USA) at a concentration of 5 × 10^6^ beads/mL. All wells contained 0.5 µg/mL of anti-CD40 (Miltenyi Biotec, Bergisch Gladbach, Germany), and the peptide-stimulated wells also contained 1 µg/mL of FastImmune anti-CD28/49d (BD Biosciences, Franklin Lakes, NJ, USA). The unstimulated (vehicle) control contained R10 with 0.4% DMSO (MilliporeSigma Canada Ltd., Oakville, ON, Canada).

Following stimulation, plates were centrifuged and the culture supernatants were collected and stored at −80°C. Cells were stained for flow cytometry using the panel in **Supplementary Table 1**. Briefly, following incubation with Zombie UV Fixable Viability dye (BioLegend, San Diego, CA, USA) and 5μg/ml of Fc Block (BD Biosciences, Franklin Lakes, NJ, USA) for 15 min at room temperature, cells were stained for chemokine receptors (CCR7, CXCR5, CCR6, CXCR3, CCR4) for 20 min at 37°C and then the remaining surface markers for 30 min at 4°C. Following staining, cells were fixed with 0.5% PFA for 30 min at 4°C and then resuspended in stain buffer for acquisition. Data were acquired using a high-throughput sampler on a BD FACSymphony A5 (BD Biosciences, Franklin Lakes, NJ, USA) using FACSDiva software v9.1. Data were analyzed with FlowJo v10.9.0.

The flow cytometry gating strategy is shown in **Supplementary Figure 1**. Memory T cells were first identified by exclusion of naive (CD45RA^+^ CCR7^+^) cells from CD4^+^ and CD8^+^ T-cell populations. Activation-induced marker (AIM)^+^ memory T cells were then defined by co-expression of CD69 and 4-1BB on CD8^+^, OX40 and 4-1BB on CD4^+^, and OX40 and CD40L on CXCR5^+^ cTfh cells (20). AIM^+^ responses are expressed as % within the indicated subset following stimulation, after subtracting the % of AIM^+^ cells in the unstimulated condition. AIM^+^ memory CD4^+^ and CD8^+^ T cells were further analyzed for memory subsets (CD45RA and CCR7), and CD4^+^ Th subsets were identified by chemokine receptor expression (CXCR5, CXCR3, CCR4, CCR6). Further characterization of AIM^+^ cells for the responding cell phenotype was restricted to samples in which the AIM response was >0.1% of the T-cell subset, and there were at least 30 AIM^+^ events available for phenotyping.

### Measurement of cytokines using the MILLIPLEX assay

Supernatants collected from unstimulated, Spike-, and CEFX-stimulated PBMCs were diluted 1:10 in phosphate buffered saline (PBS) and analyzed using the MILLIPLEX Human CD8^+^ T-Cell Magnetic panel (14-plex) kit (MilliporeSigma Canada Ltd., Oakville, ON, Canada) for concentrations of granulocyte-macrophage colony-stimulating factor (GM-CSF), interferon (IFN)-γ, interleukin (IL)-10, granzyme A, IL-13, granzyme B, IL-2, IL-4, IL-5, IL-6, macrophage inflammatory protein (MIP)-1α, MIP-1β, tumor necrosis factor (TNF)-α, and perforin. An in-house culture control medium of 10% R10 in PBS was prepared as matrix solution for all standards and quality controls, and kits were utilized according to the manufacturer’s instructions.

Data were acquired on a BioPlex 200 System with BioPlex Software Manager V.5 (MilliporeSigma Canada Ltd., Oakville, ON, Canada), and data analysis was performed with Millipore Belysa Immunoassay Curve Fitting Software V1.2. For concentration falling below the kit-specified analyte-specific minimum detectable concentration (MDC), results were reported as one-half MDC. Interassay reproducibility was assessed based on consistency of standard curve parallelism and slope values were reported by Belysa analysis software. Intra-assay performance was assessed based on calculated concentrations of quality controls and sample replicate %CV.

### Statistical analysis

Longitudinal phenotypic data across three or more groups or time points were compared using the Kruskal–Wallis non-parametric test, followed by Dunn’s multiple comparisons pairwise post-test where applicable. Comparisons involving two groups were performed by the Mann–Whitney *U* test, with correction for multiple comparisons using the Holm–Sidak method. Paired comparisons of two time points (e.g., pre- and post-boost) were conducted using the Wilcoxon matched-pairs signed rank test. Correlations between parameters were assessed using the Spearman test. GraphPad Prism software, version 10.0.3 was used for statistical analysis.

### Study approval

This study received ethical approvals from the University of Manitoba Health Research Ethics Board and the Health Canada-Public Health Agency of Canada Research Ethics Board. The study participants provided informed consent prior to participation.

## Results

### Participant characteristics

A total of 70 individuals who received at least two doses of the BNT162b2 COVID-19 vaccine were included in this study. Of these, 60 individuals also received a third dose of an mRNA-based vaccine over the course of follow-up. The demographic and clinical characteristics of the study participants are shown in **Table 1**. The standard and extended dose interval groups had comparable distributions in age, sex, and all clinical characteristics. Biological sex had no impact on any immune outcomes measured (**Supplementary Figure 2**).

**Table 1:** Demographic and clinical characteristics of the study participants.

Characteristic	Dose interval group^a^		*p*-value^a^
	Standard (*n* = 41)	Extended (*n* = 29)	
Demographics			
Age at enrollment	44 (35–50.5)	43 (34–47)	0.75
Female sex	26 (63.4)	20 (69.0)	0.80
BMI (kg/m**^2^**)	24.40 (21.25–30.25)	26.70 (23.08–30.43)	0.24
Medical history			
History of cancer	2 (4.88)	0 (0.00)	0.51
Diabetes	1 (2.44)	0 (0.00)	>0.99
Cardiovascular disease (total)	3 (7.32)	4 (13.79)	0.44
Hypertension	3 (7.32)	3 (10.34)	0.69
History of stroke	0 (0.00)	1 (3.45)	0.41
Asthma/allergy	7 (17.07)	5 (17.24)	>0.99
Lung disease	0 (0.00)	1 (3.45)	0.41
Subsequent COVID-19 infection reported	26 (76.5)	17 (77.3)	>0.99
Current medication			
PPI	6 (14.63)	2 (6.90)	0.46
SSRI/SNRI	3 (7.32)	7 (24.14)	0.08
ACE inhibitors	2 (2.44)	2 (6.90)	0.57
Alpha/beta blockers	2 (4.88)	1 (3.45)	>0.99
Steroid	4 (9.76)	2 (6.90)	>0.99
Bronchodilators	1 (2.44)	1 (3.45)	>0.99
Statins	1 (2.44)	2 (6.90)	0.57
Biguanides	1 (2.44)	0 (0.00)	>0.99

a Continuous variables expressed as median (IQR). Categorical variables expressed as *n* (% of group).

b Continuous variables were tested for normal distribution and then groups were compared by the *t*-test or Mann–Whitney test as appropriate. For categorical variables, groups were compared by Fisher’s exact test.

**Figure 1:**
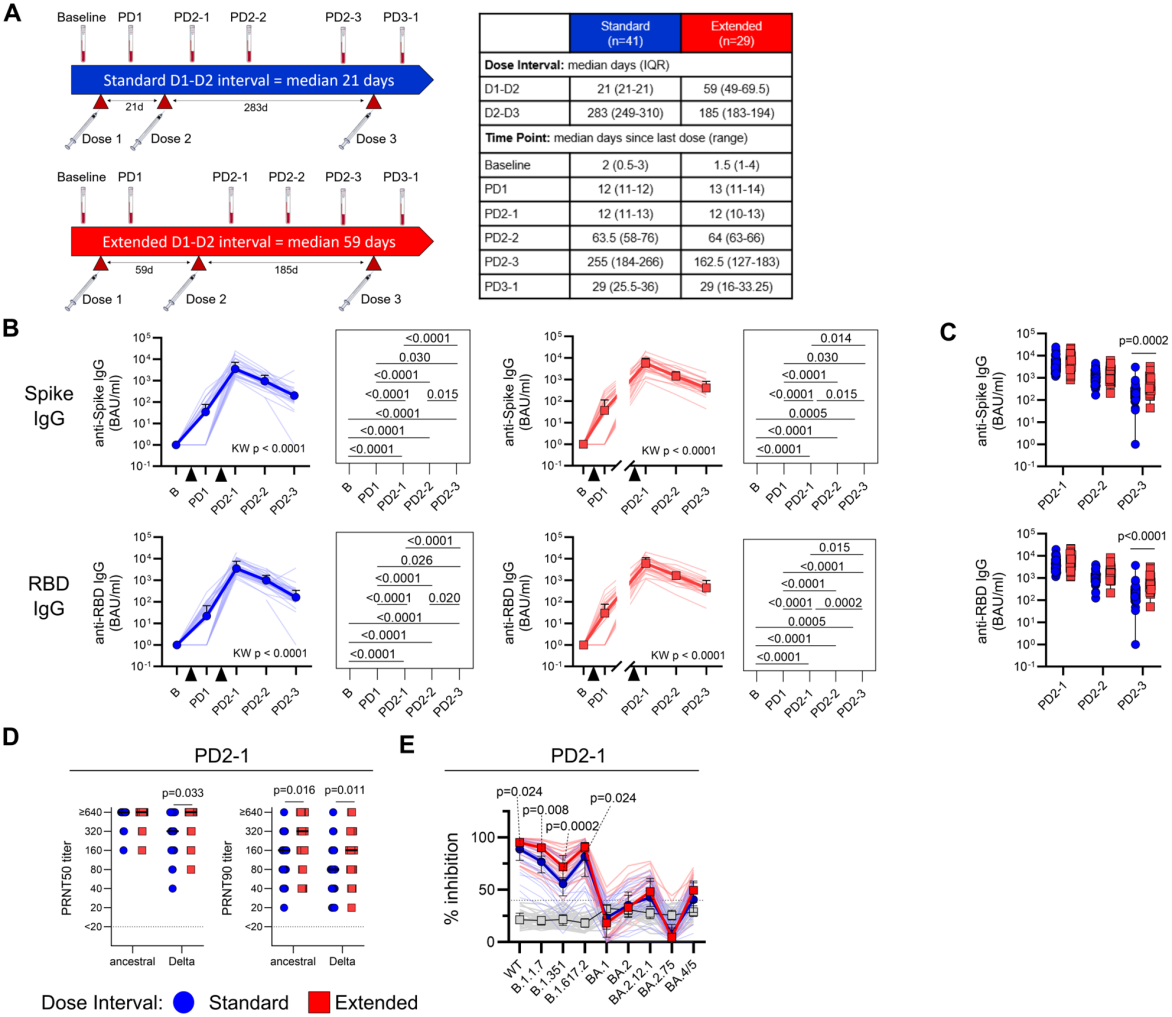
An extended primary dose interval improves humoral responses to BNT162b2 COVID-19 vaccine. **(A)** Timeline of vaccine dose administration and blood sampling. Dose 2 was administered a median of 21 days post-dose 1 for the standard interval group (*n* = 41) or 59 days (IQR 49–69.5 days) post-dose 1 for the extended interval group (*n* = 29). Blood was collected before vaccination (baseline) and then at time points after the first (PD1), second (PD2-1, PD2-2, PD2-3), and third (PD3-1) vaccine dose, with median days (IQR) relative to the last dose for each sample shown in the table. **(B)** Trajectory of anti-Spike (top) and anti-RBD (bottom) IgG titers for the standard (blue circles) and extended (red squares) dose interval groups. Time points were compared using the Kruskal–Wallis test with Dunn’s post-test for pairwise comparisons. **(C)** Comparison of anti-Spike and anti-IgG titers between the standard and extended dose interval groups at time points following dose 2 (Mann–Whitney tests with Holm–Sidak correction). **(D)** Comparison of PRNT50 and PRNT90 titers to ancestral or Delta/B.1.617.2 SARS-CoV-2 between standard and extended dose interval groups at PD2-1 time point (Mann–Whitney tests with Holm–Sidak correction). **(E)** Comparison of inhibition of ACE2 binding to variant RBDs by plasma from standard and extended dose interval participants at PD2-1 (Mann–Whitney tests with Holm–Sidak correction). Bold lines in line graphs in **(B)** and **(E)** show median values. Box plots in **(C)** depict median and IQR; whiskers depict range.

Of the 70 participants, 41 had a standard interval of 21 days and 29 participants had an extended interval (median 59 days, IQR 49–69.5) between vaccine doses 1 and 2. Blood samples were collected prior to vaccination and then a median of 12 days after dose 1 (PD1); 12 days (PD2-1), 64 days (PD2-2), and 184 days (PD2-3) after dose 2; and 29 days after dose 3 (PD3-1). Details of the vaccination doses and sample collection time points are shown in **Figure 1A** and **Table 2**.

**Table 2:** Vaccine dose information and sampling schedule.

	Dose interval group^a^		*p*-value^a^
	Standard (*n* = 41)	Extended (*n* = 29)	
Vaccine doses			
Dose 1–dose 2 interval (days)	21 (21–21)	59 (49–69.5)	<0.00001
Dose 2–dose 3 interval (days)	283 (249–310)	185 (183–194)	<0.00001
Vaccine regimen			
Dose 1 reported	41 (100)	29 (100)	>0.99
Pfizer BNT162b2	41 (100)	29 (100)	>0.99
Dose 2 reported	41 (100)	29 (100)	>0.99
Pfizer BNT162b2	41 (100)	29 (100)	>0.99
Dose 3 reported	35 (85.4)	25 (86.2)	>0.99
Pfizer BNT162b2	19 (54.3)	12 (48.0)	0.79
Moderna mRNA1273	6 (17.1)	2 (8.0)	0.45
Moderna Spikevax Bivalent (BA.1)	1 (2.86)	0 (0.0)	>0.99
Unsure	9 (25.7)	11 (44.0)	0.17
Sample collection visits			
Baseline (days pre-dose 1)	2 (0.5–3)	1.5 (1–4)	0.78
PD1 (days post-dose 1)	12 (11–12)	13 (11–14)	0.19
PD2-1 (days post-dose 2)	12 (11–13)	12 (10–13)	0.94
PD2-2 (days post-dose 2)	63.5 (58–76)	64 (63–66)	0.94
PD2-3 (days post-dose 2)	255 (184–266)	162.5 (127–183)	<0.00001
PD2-3 (days pre-dose 3)	51 (18–68)	20 (1–58)	0.10
PD3 (days post-dose 3)	29 (25.25–36)	29 (16–33.25)	0.38

a Continuous variables expressed as median (IQR). Categorical variables expressed as *n* (% of group).

b Continuous variables were tested for normal distribution and then groups were compared by the *t*-test or Mann–Whitney test as appropriate. For categorical variables, groups were compared by Fisher’s exact test.

### An extended dose interval is associated with improved antibody durability and breadth

The study participants with both standard and extended dose intervals mounted robust plasma anti-S1 and anti-RBD IgG responses after doses 1 and 2 (*p* < 0.0001 for both groups, **Figure 1B**). As expected, groups did not differ by the number of responders or antibody titers after the first dose, before their vaccination schedules diverged (**Supplementary Figure 3A**). Antibody titers at the first sample collected after dose 2 (PD2-1) did not differ between the two groups, but by 6–8 months post-dose 2 (PD2-3), participants with an extended interval retained higher titers of anti-S1 (*p* = 0.0002) and anti-RBD (*p* < 0.0001) antibodies, suggesting slower rates of decline of circulating antibodies in this group (**Figure 1C**). We also categorized participants as high or low antibody responders according to whether their antibody titers were above (high) or below (low) the median. A greater proportion of the participants with an extended dose interval were found to be high responders at PD2-3, with a similar trend observed at PD2-1 (**Supplementary Figure 3B**).

Viral neutralization capacity of plasma antibodies also differed between the standard and extended dose interval groups. Neutralizing antibodies against ancestral and Delta SARS-CoV-2 isolates were first assessed using live-virus PRNT assays (standard *n* = 33, extended *n* = 25). PRNT50 and PRNT90 titers represent the maximum dilution at which 50% and 90% inhibition of viral replication was observed, respectively. At PD2-1 (10–14 days), the extended interval group exhibited significantly higher PRNT90 titers against ancestral virus (*p* = 0.016). This difference was more pronounced for the Delta variant, with the extended interval group showing significantly higher PRNT50 (*p* = 0.033) and PRNT90 (*p* = 0.011) neutralizing antibody titers compared to the standard interval group (**Figure 1D**).

The assessment of plasma neutralizing antibodies was extended to evaluate the response against other SARS-CoV-2 variants. For this purpose, PD2-1 plasma samples from the standard (*n* = 30) or extended (*n* = 26) interval groups were analyzed using an ACE2 binding inhibition assay, in which antibodies binding RBD variants block the interactions with ACE2. Assessment of inhibition of ACE2 binding to variant versions of RBD demonstrated better inhibition of the ancestral/wild-type (WT) (*p* = 0.024), Alpha/B.1.1.7 (*p* = 0.008), Beta/B.1.351 (*p* = 0.0002), and Delta/B.1.617.2 (*p* = 0.024) variants in plasma samples with an extended interval compared to the standard interval group at PD2-1 (**Figure 1E**), consistent with PRNT data. Although many participants demonstrated detectable, but lower inhibition of Omicron sublineages BA.1, BA.2, BA2.12.1, BA.2.75, and/or BA.4/5, extending the dose interval did not show any discernable effect on Omicron inhibition when compared to standard dosing (**Figure 1E**).

### Dose interval does not affect the development of antigen-specific T-cell responses

**Figure 2:**
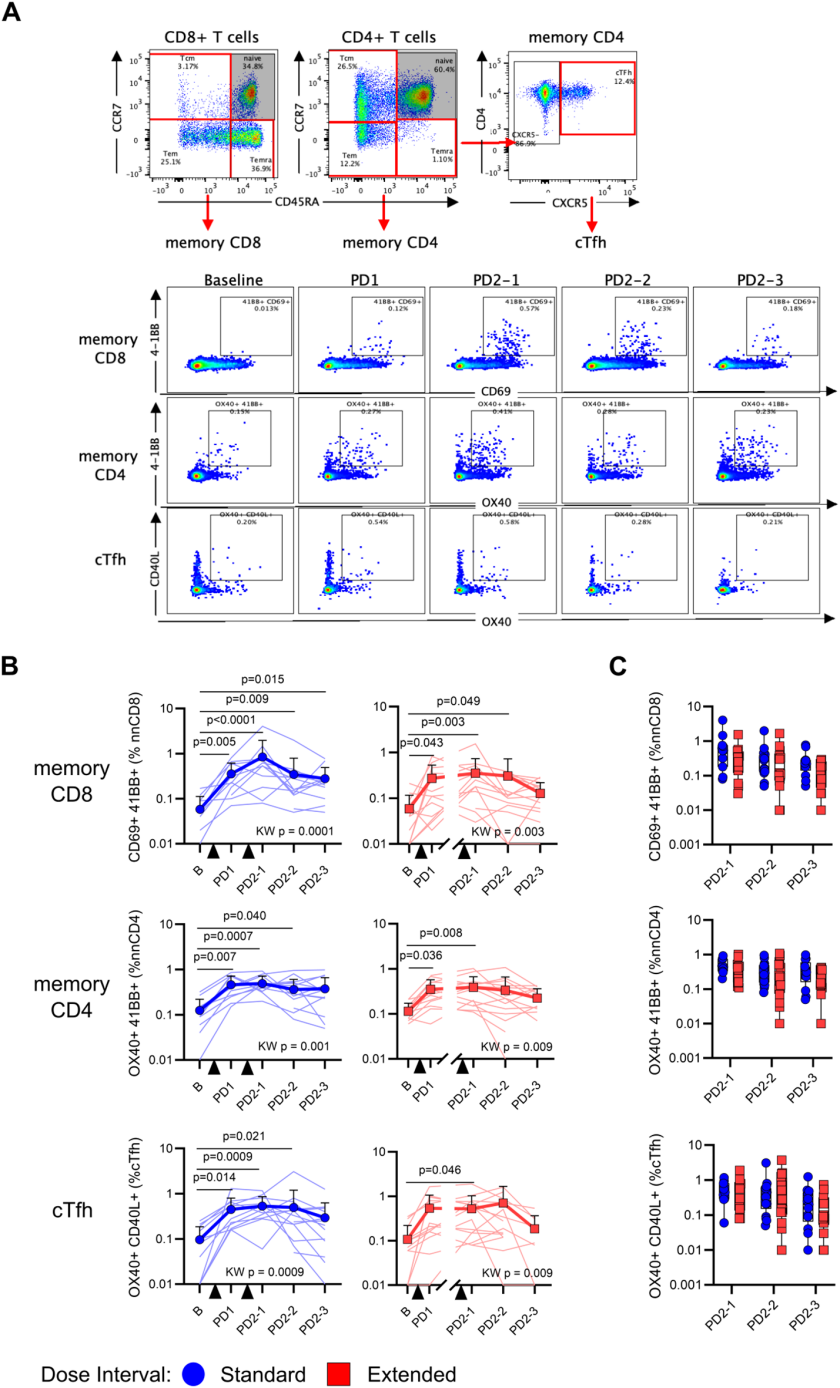
An extended primary dose interval does not impact the development of Spike-specific memory T-cell responses. **(A)** Representative staining of memory T cells following stimulation with SARS-CoV-2 Spike peptides. Naive CD8^+^ and CD4^+^ T cells were excluded and then memory T cells were evaluated for the co-expression of AIM markers 4-1BB and CD69 (CD8), 4-1BB and OX40 (CD4), or CD40L and OX40 (CXCR5^+^ CD4^+^ cTfh). **(B)** Trajectory of Spike-specific memory CD8^+^ (top), CD4^+^ (middle), or cTfh (bottom) AIM responses for the standard (blue circles) and extended (red squares) dose interval groups. Time points were compared using the Kruskal–Wallis test with Dunn’s post-test for pairwise comparisons. **(C)** Comparison of memory CD8^+^ (top), CD4^+^ (middle), and cTfh (bottom) T-cell AIM responses between standard and extended dose interval groups at time points following dose 2 (Mann–Whitney tests with Holm–Sidak correction). Bold lines in line graphs in **(B)** show median values. Box plots in **(C)** depict median and IQR; whiskers depict range.

Memory T-cell responses were assessed using the AIM assay, where antigen-specific memory CD8^+^ T-cell responses were defined by co-expression of CD69 and 4-1BB, CD4^+^ T-cell responses by OX40 and 4-1BB, and circulating T follicular helper (cTfh) responses by OX40 and CD40L (**Figure 2A**, **Supplementary Figure 1**). As shown in **Figure 2B**, vaccination elicited significant increases in SARS-CoV-2 Spike-specific memory CD8^+^ (standard *p* = 0.0001, extended *p* = 0.003), CD4^+^ (standard *p* = 0.001, extended *p* = 0.009), and cTfh (standard *p* = 0.0009, extended *p* = 0.009) responses in both groups, relative to baseline. Comparisons of responses between time points showed that, although AIM^+^ memory T cells assessed after the first and second vaccine doses were significantly elevated relative to baseline, post-vaccine time points did not significantly differ from each other, suggesting relative stability in memory T-cell responses (**Figure 2B**). The proportions of AIM^+^ memory T cells did not differ between participants with standard and extended dose intervals at any time point following the second vaccine dose (**Figure 2C**).

**Figure 3:**
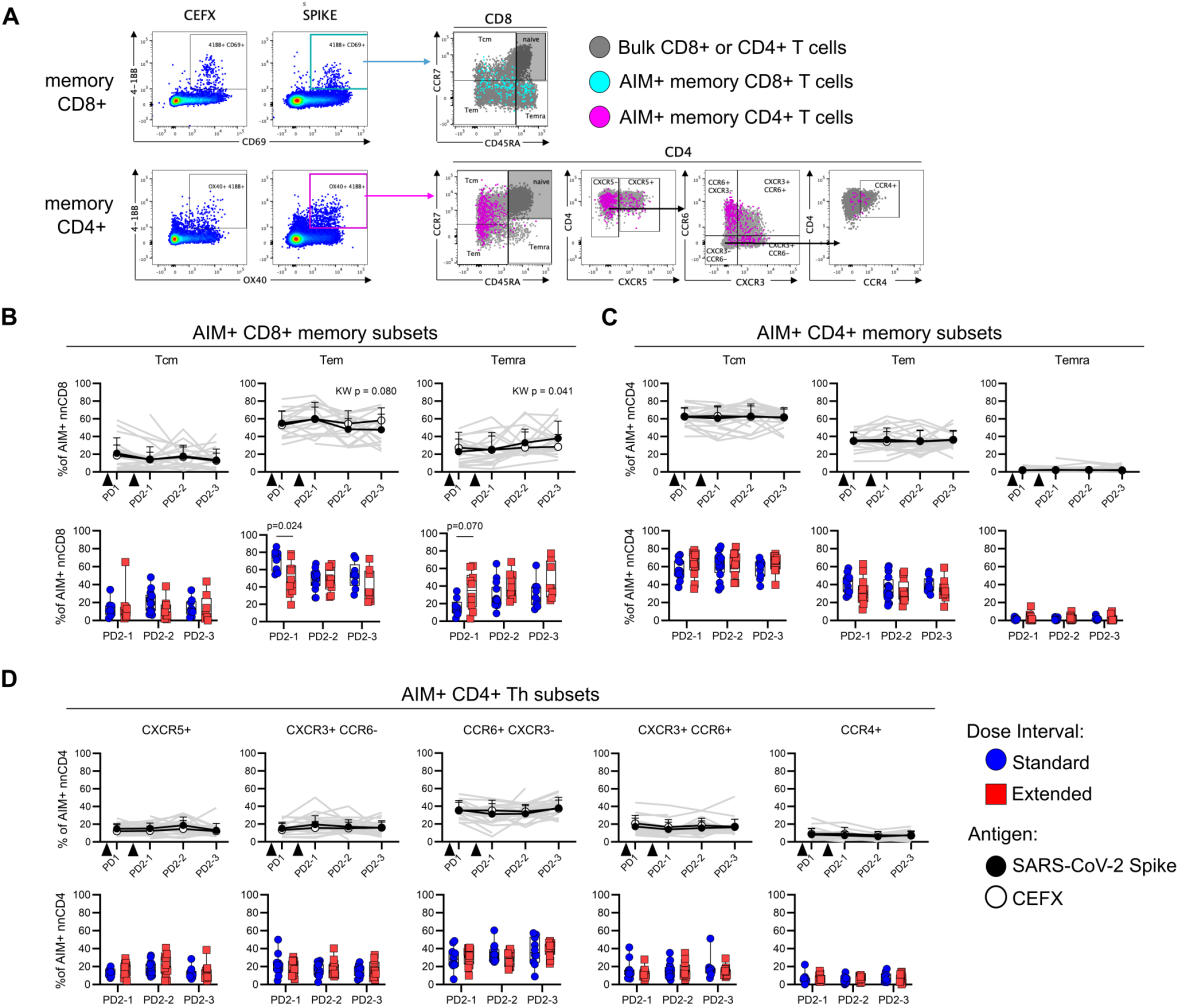
Dose interval does not impact the phenotype of Spike-specific memory T cells. **(A)** Representative gating of memory (CD45RA/CCR7) subsets within Spike-specific nnCD8^+^ and nnCD4^+^ T-cell populations and Th subsets as defined by the expression of CXCR5, CCR6, CXCR3, and CCR4. **(B, C)** Trajectory of CD8^+^ **(B)** or CD4^+^ **(C)** Spike-specific (filled circles) and CEFX-specific (open circles) Tcm, Tem, and Temra subsets among AIM^+^ cells (top panel; Kruskal–Wallis test). Bottom panel shows comparison of Spike-specific memory CD8^+^ **(B)** or CD4^+^ **(C)** T-cell subsets between the standard and extended dose interval groups at time points following dose 2 (Mann–Whitney tests with Holm–Sidak correction). **(D)** Trajectory of CD4^+^ Spike-specific (filled circles) and CEFX-specific (open circles) T helper subsets as defined by chemokine receptor expression (top panel; Kruskal–Wallis test). The bottom panel shows comparison of Spike-specific CD4^+^ T helper subsets between the standard and extended dose interval groups at time points following dose 2 (Mann–Whitney tests with Holm–Sidak correction). Bold lines in line graphs in **(B–D)** show median values. Gray lines show individual Spike responses. Box plots in **(B–D)** depict median and IQR; whiskers depict range.

Antigen-specific AIM^+^ memory T cells were further categorized into central (Tcm), effector (Tem), and CD45RA-expressing effector (Temra) memory subsets based on differential expression of CD45RA and CCR7 (**Figure 3A**). Among AIM^+^ Spike-specific memory CD8^+^ T cells, there was a modest but significant increase in Temra (*p* = 0.041) and a concomitant trend toward a decrease in Tem (*p* = 0.080) following vaccination (**Figure 3B**). In light of these temporal changes, the ratios of antigen-specific memory subsets to the proportions of these subsets among bulk CD8^+^ T cells were analyzed, revealing a gradual shift toward a Temra phenotype in the months following dose 2 (**Supplementary Figure 4**). At PD2-1, the extended interval group had significantly fewer Tem (*p* = 0.024) and a trend toward more Temra (*p* = 0.07) compared to the standard interval group (**Figure 3B**). In contrast to CD8^+^ T cells, the memory phenotypes of AIM^+^ Spike-specific memory CD4^+^ T cells remained stable over time and did not differ between groups (**Figure 3C**). Spike-specific AIM^+^ memory CD4^+^ T cells were further phenotyped into Th-like subsets based on differential expression of chemokine receptors: CXCR5^+^ (cTfh-like), CXCR5^−^ CCR6^+^ CXCR3^−^ (Th17-like), CXCR5^−^ CCR6^−^ CXCR3^+^ (Th1-like), CXCR5^−^ CCR6^+^ CXCR3^+^ (Th1/17-like), and CXCR5^−^ CCR6^−^ CXCR3^−^ CCR4^+^ (Th2-like) (**Figure 3A**), as previously described by others (21, 22). Spike-specific Th subsets did not change over time and were not affected by dose interval (**Figure 3D**). As expected, phenotypes of AIM^+^ CEFX-specific CD8^+^ and CD4^+^ T cells remained stable over time (**Figures 3B–D**, **Supplementary Figure 4**).

**Figure 4:**
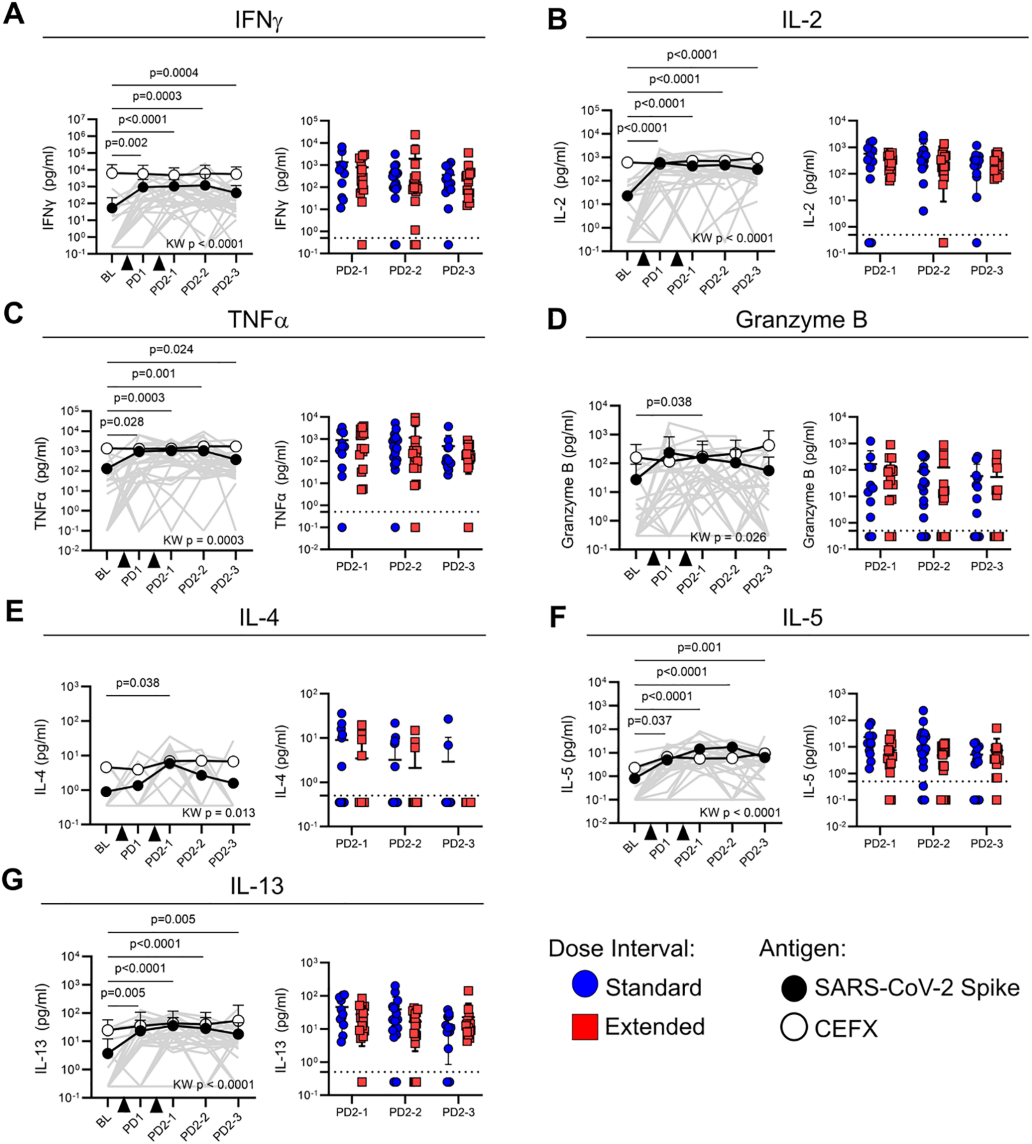
Dose interval does not impact cytokine production by Spike-specific memory T cells. Line graphs show the production of **(A)** IFN-γ, **(B)** IL-2, **(C)** TNF-α, **(D)** granzyme B, **(E)** IL-4, **(F)** IL-5, and **(G)** IL-13 in response to Spike (closed circles) or CEFX (open circles) peptide stimulation at various time points post-vaccination. Time points were compared using the Kruskal–Wallis test with Dunn’s post-test for pairwise comparisons, with *p*-values for Spike-specific responses shown. Bold lines in line graphs show median values. Gray lines show individual Spike responses. Scatter box plots show comparisons of Spike-specific cytokine responses between the standard and extended dose interval groups at time points following dose 2 (Mann–Whitney tests with Holm–Sidak correction). Box plots depict median and IQR; whiskers depict range. Dotted line indicates cutoff.

To evaluate the functional profile of antigen-specific memory T cells, cytokine production in response to peptide stimulation was assessed in cell culture supernatants. Concentrations of IFN-γ (*p* < 0.0001), IL-2 (*p* < 0.0001), TNF-α (*p* = 0.0003), granzyme B (*p* = 0.026), IL-4 (*p* = 0.013), IL-5 (*p* < 0.0001), and IL-13 (*p* < 0.0001) were all significantly higher in Spike-stimulated supernatants from post-vaccine time points relative to baseline (**Figures 4A–G**). The levels of IFN-γ, IL-2, TNF-α, IL-5, and IL-13 following Spike stimulation were sustained at all post-vaccination time points tested (**Figures 4A–C, F, G**). In contrast, granzyme B and IL-4 were only significantly higher than baseline at PD2-1 (*p* = 0.028 and *p* = 0.044, respectively) (**Figures 4D, E**). Cytokine levels in response to Spike stimulation did not differ between standard and extended dose intervals (**Figures 4A–G**). Cytokine concentrations in CEFX-stimulated culture supernatants remained stable over time (**Figures 4A–G**).

### A third dose of vaccine abrogates the impact of primary dose interval on antibody responses

**Figure 5:**
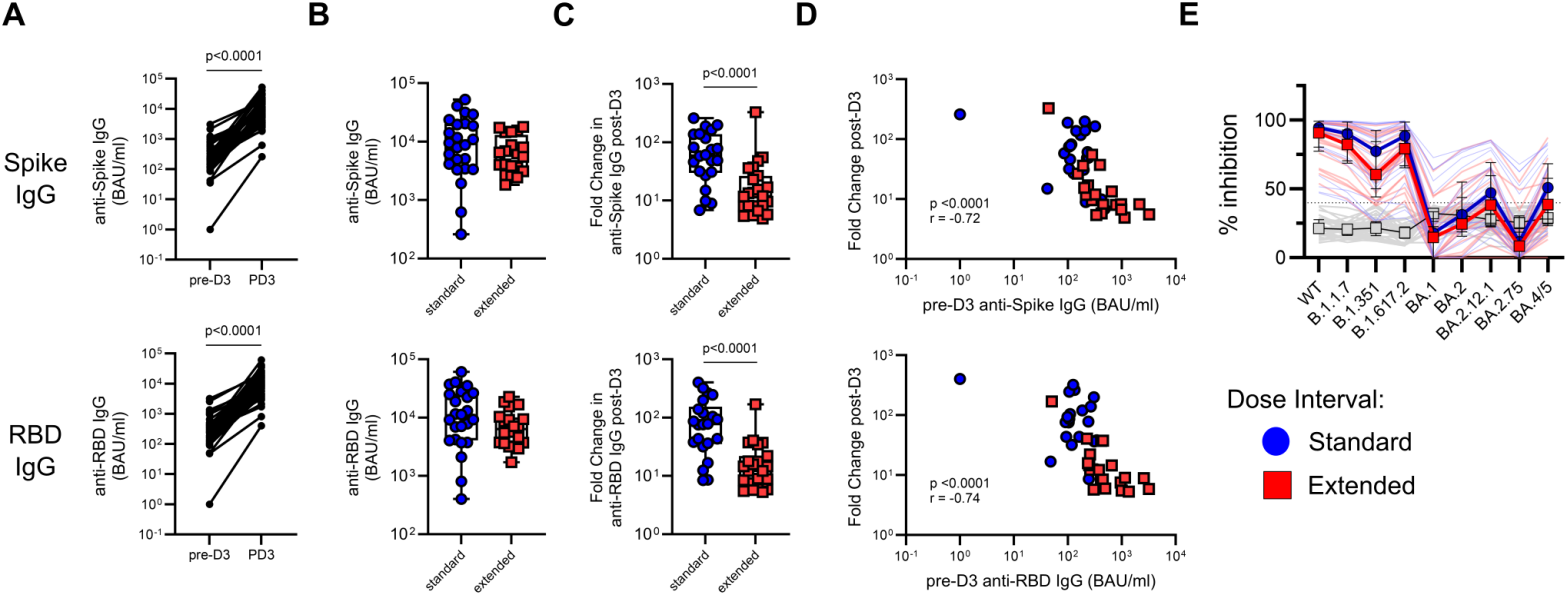
A third vaccine dose boosts SARS-CoV-2-specific antibody responses irrespective of primary dose interval. **(A)** Comparison of matched pre- and post-dose 3 titers of anti-Spike and anti-RBD IgG (Wilcoxon test). **(B, C)** Comparison of antibody titers **(B)** and fold change in antibody titers **(C)** following dose 3 between the standard and extended dose interval groups (Mann–Whitney tests). **(D)** Correlation between pre-boost titers of anti-Spike or anti-RBD IgG and fold change in respective titers following dose 3. **(E)** Comparison of inhibition of ACE2 binding to variant RBDs by plasma from standard and extended dose interval participants at PD3-1 diluted either 1:50 or 1:100, as indicated (Mann–Whitney tests with Holm–Sidak correction). Gray lines indicate baseline negative control plasmas. Box plots in panels **(B, C)** depict median and IQR; whiskers depict range. Bold lines in the line graph in panel **(E)** show median values.

Participants in both the standard and extended dose interval groups became eligible for a third (booster) dose at the same time, which was approximately 8–10 months post-dose 2 for the standard interval group and 6 months post-dose 2 for the extended interval group (**Figure 1A**, **Table 2**). Samples collected post-dose 3 were available for analysis from 24 participants in the standard interval group and 20 participants in the extended interval group, with matched pre-dose 3 samples (i.e., PD2-3) available for 22 and 20 of those participants, respectively. A third vaccine dose significantly boosted plasma IgG targeting Spike (*p* < 0.0001) and RBD (*p* < 0.0001) (**Figure 5A**). Antibody titers did not differ between groups post-dose 3 (**Figure 5B**), but the fold change following the booster was higher for the standard interval group (*p* < 0.0001 for both Spike and RBD; **Figure 5C**). Since post-dose 3 antibody titer did not differ between groups, this was likely driven by lower pre-dose titers observed in the standard interval group at PD2-3 (**Figure 1C**). Indeed, the fold change in anti-Spike or anti-RBD IgG titers correlated inversely with pre-dose 3 titers (Spike *p* < 0.0001, *r* = −0.72, RBD *p* < 0.0001, *r* = −0.74; **Figure 5D**).

To evaluate antibody neutralization potential, a subset of post-dose 3 samples (standard *n* = 22, extended *n* = 21) was analyzed using the ACE2 binding inhibition assay. In contrast to what was observed following the second dose, participants with standard and extended primary dose intervals demonstrated comparable inhibition of ACE2 binding to RBD from all variants tested (**Figure 5E**).

### A third dose of vaccine increases memory CD8^+^ T-cell responses independent of dose interval

**Figure 6:**
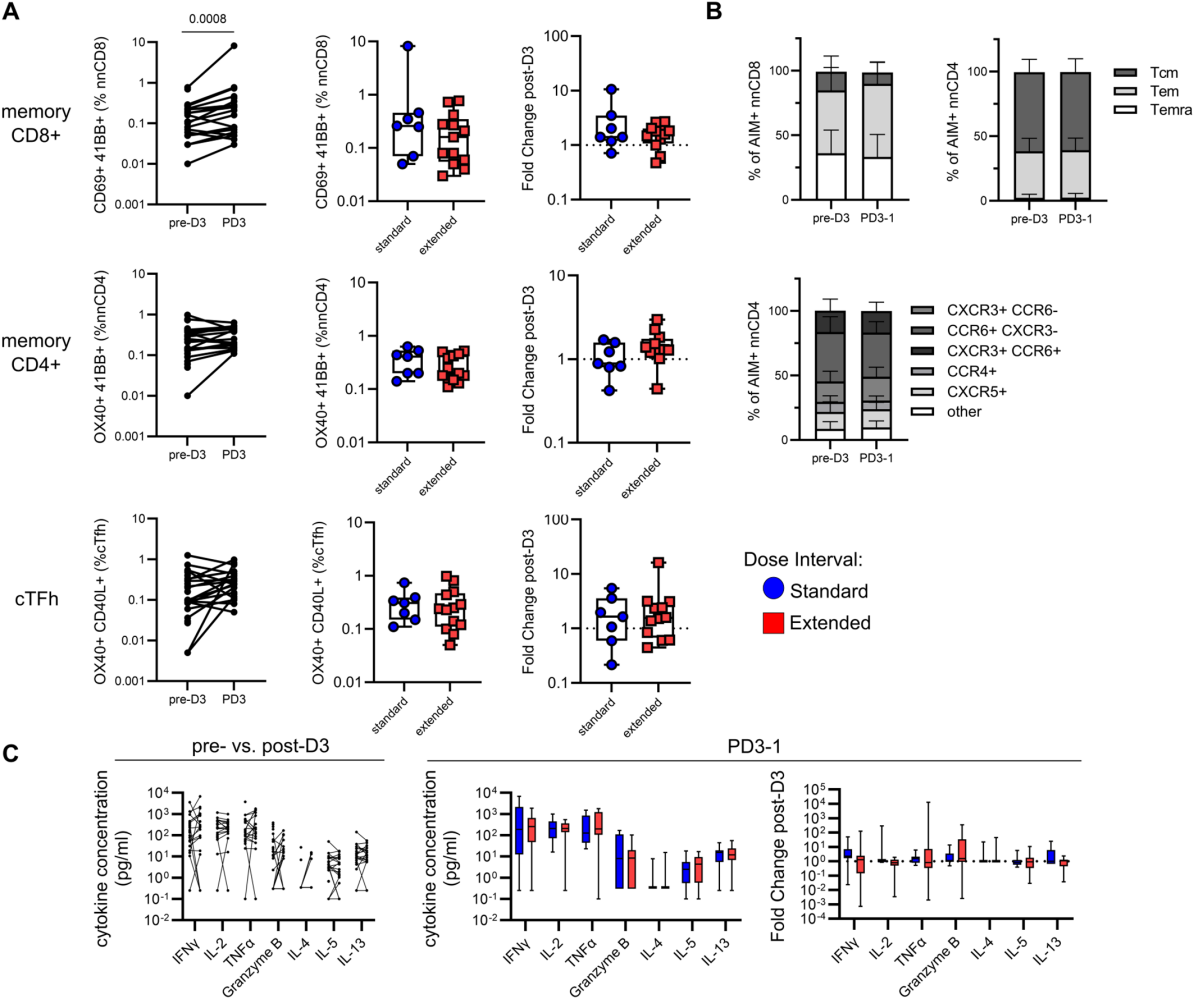
A third vaccine dose has minimal impact on Spike-specific T-cell responses irrespective of primary dose interval. **(A)** Comparison of matched pre- and post-dose 3 Spike-specific memory CD8^+^, CD4^+^ T-cell and cTfh responses (Wilcoxon test). Box plots show comparisons of AIM^+^ memory CD8^+^, CD4^+^ T cells, and cTfh cells and fold change in responses following dose 3 between the standard and extended dose interval groups (Mann–Whitney tests). **(B)** Comparison of Spike-specific memory CD8^+^ or CD4^+^ T-cell subsets and CD4^+^ T helper subsets between matched pre- and post-dose 3 samples (Mann–Whitney tests with Holm–Sidak correction). **(C)** Comparison of cytokine responses in response to Spike peptide stimulation in matched pre- and post-dose 3 samples. Box plots show comparisons of cytokine concentrations and fold change in cytokine responses following dose 3 between the standard and extended dose interval groups (Mann–Whitney tests with Holm–Sidak correction). Box plots in panels **(A, C)** depict median and IQR; whiskers depict range.

SARS-CoV-2 Spike-specific memory CD8^+^ T-cell responses increased following the third dose in participants from both groups with matched pre- and post-boost samples (*p* = 0.0008, **Figure 6A**). This increase was independent of dose interval, with no differences observed between groups in the proportion of post-boost Spike-specific memory CD8^+^ T cells or the magnitude of the fold change following the third dose (**Figure 6A**). This similarity between groups in the post-dose 3 CD8^+^ T-cell response is expected, since the two groups had comparable responses to the second dose and experience similar declines in the population of antigen-specific T cells (**Figures 1B, C**). In contrast to CD8^+^ T cells, the third dose did not impact Spike-specific memory CD4^+^ T cell or cTfh cell responses, regardless of dose interval (**Figure 6A**).

The profiles of memory subsets among responding Spike-specific CD8^+^ and CD4^+^ T cells were compared before and after the third dose. The distributions of the Tcm, Tem, and Temra subsets among AIM^+^ CD8^+^ and CD4^+^ T cells were not affected by the third dose (**Figure 6B**) and the dynamic changes in Spike-specific CD8^+^ memory subsets stabilized after dose 3 (**Supplementary Figure 4**). Similarly, Th-like subsets among AIM^+^ CD4^+^ T cells were comparable pre- and post-dose 3 (**Figure 6B**). Despite an increase in the percentage of AIM^+^ memory CD8^+^ T cells, no differences were observed in the amount of IFN-γ, IL-2, TNF-α, granzyme B, IL-4, IL-5, or IL-13 detected in culture supernatants in pre- and post-dose 3 samples (**Figure 6C**). Dose interval was not associated with the levels of cytokines detected or the magnitude of fold change following the third dose (**Figure 6C**). Collectively, these data indicate that a third dose may lead to some expansion in the number of antigen-specific CD8^+^ T cells but does not impact their cytokine production, regardless of the interval of time between doses.

## Discussion

This study reports that individuals who had an extended interval between their first two doses of BNT162b2 demonstrated improved binding antibody durability and better neutralizing antibody magnitude and breadth against closely related SARS-CoV-2 variants compared to those with a standard 3-week interval. However, this increase in breadth was insufficient to improve the relatively weak inhibition of Omicron sublineages observed after two doses, demonstrating the limited utility of adjusting dose intervals to protect against distantly related variants. Dose interval did not have a discernible impact on SARS-CoV-2-specific memory T-cell responses. Furthermore, this study demonstrated that differences in immune outcomes resulting from a delayed second dose are overcome by a third (booster) dose of an mRNA-based vaccine.

Our data are consistent with other reports showing a positive impact of extended dose intervals on antibody responses to SARS-CoV-2 and neutralization of variants (8–14). Although both groups had similar antibody titers early after dose 2, individuals with an extended dose interval exhibited a slower rate of decline compared to those with the standard interval. Some previous cross-sectional studies evaluated antibody titers at later time points after the second dose (8–11, 13), which many explain why the extended dose interval was associated with higher titers in those study populations (23, 24).

We used two methods to evaluate neutralizing antibodies. Using the live-virus PRNT assay, we showed that an extended dose interval resulted in higher neutralizing antibody titers against the ancestral strain and even more pronounced improvement against the Delta variant. This improved functionality was evident even when absolute antibody titers did not differ between groups, suggesting differences in antibody affinity and avidity resulting from B-cell maturation (8–10, 12, 25).

The observation that dose interval impacted neutralization of the Delta variant to a greater extent than the ancestral strain led us to expand our analysis to additional variants. For this, we used an ACE2 binding inhibition assay to assess the ability of plasma antibodies to block the binding of ACE2 to variant RBD proteins. The extended dose interval was associated with better inhibition of the ancestral, Alpha/B.1.17, Beta/B.1.351, and Delta/B.1.617.2 variants, consistent with other studies (8–10, 12). Inhibition of ACE2 binding to RBD proteins from Omicron sublineages BA.1, BA.2, BA.2.12.1, BA2.75, and BA.4/5 was considerably reduced compared to ancestral and pre-Omicron variants, but inhibition was nevertheless observed in many participants. However, dose interval did not have any impact on neutralization of Omicron variants, indicating that the benefit of an extended dose interval is limited and does not confer protection against variants that are divergent from the original antigenic exposure. This contrasts with one study that found that a longer primary dose interval was associated with improved neutralization of an Omicron/BA.1 Spike-bearing pseudovirus (25–28). To our knowledge, our study is the first to evaluate the impact of the primary dose interval of mRNA-based COVID-19 vaccines on cross-recognition of multiple Omicron sublineages.

The observed improvement in antibody breadth is likely due to the evolution of B-cell responses in germinal centers, where B cells undergo somatic hypermutation, resulting in memory B cells with higher affinity and broader specificity for antigenic variants (29, 30). Indeed, post-dose 2 memory B cells analyzed from individuals with an extended dose interval have previously been observed to be phenotypically more mature than those from people with a standard dose interval (15), consistent with memory B-cell evolution over months following SARS-CoV-2 infection (31, 32) or vaccination (33–35) and enhanced antibody neutralization potency and breadth at later time points (32, 36). Analysis of antibody sequences has shown that while some mutations accrued during SHM conferred higher affinity for the ancestral Spike and closely related variants, additional mutations were necessary for neutralization of Omicron (37). This may explain why our data showed that longer dose intervals were associated with improved inhibition of Alpha, Beta, and Delta, but not Omicron.

A third dose of vaccine resulted in a significant increase in binding antibody titers, irrespective of the interval between doses in the primary vaccine series, and there was no difference between groups in post-boost antibody titer or neutralization, consistent with previous reports (28, 38, 39). Importantly, some participants in the study received a homologous Pfizer BNT162b2 booster, while others received Moderna 1273 for their third dose. A previous work has demonstrated the positive impact of heterologous booster regimens on vaccine immunogenicity (40). Our dataset was not statistically powered to assess how heterologous boosting impacted antibody responses in the context of dose intervals, but this may be an important consideration in optimizing vaccine delivery.

Evaluation of T-cell responses demonstrated that irrespective of dose interval, vaccination led to robust Spike-specific memory CD8^+^ and CD4^+^ T cells, including cTfh cells. Memory T-cell responses exhibited a slow decline in the months following the second dose, with greater decay seen in CD8^+^ compared to CD4^+^ T cells, consistent with previous reports (36). No impact of dose interval on antigen-specific memory T-cell development was observed. This is likely due to the rapid formation of memory T-cell responses following vaccination and the stability of those responses over time (41). In our study, memory CD8^+^ T-cell responses were dominated by Tem and Temra subsets, with a smaller population of responding Tcm cells. There was a progressive decline in responding Tem and a concomitant increase in Temra among antigen-specific memory CD8^+^ T cells in the months following dose 2, consistent with memory cell differentiation (20). Participants with a short dose interval had transiently higher proportions of Tem and fewer Temra at PD2-1, in line with a shorter total duration since the first dose, and less total time for memory subset differentiation. In contrast to CD8^+^ T cells, memory CD4^+^ T-cell responses were dominated by Tcm cells and subset distribution remained stable over time, with no impact of dose interval.

Previous studies have shown that early memory CD4^+^ T-cell responses correlate with humoral outcomes at later time points following vaccination (36, 42). Given the distinct antibody responses observed in individuals with an extended dose interval, we explored whether extending the dose interval may result in differences in the T helper subset profile of using expression of chemokine receptors as surrogate markers for subset identification (21, 22). The Spike-specific response was dominated by cells expressing CCR6 (Th17-like), followed by CXCR3, either alone (Th1-like) or in combination with CCR6 (Th1/17-like), and remained stable over time. This is consistent with what was observed in some previous studies (15, 43), while others found a predominance of Th1 responses (36, 42), likely due to differences in assay conditions and panels (15). We did not observe any impact of dose interval on the Th subset distribution of memory CD4^+^ T cells that responded to SARS-CoV-2 Spike peptides. However, our analysis was limited to CD4^+^ T cells circulating in the blood. Given the importance of Tfh cells in germinal center B-cell responses, we cannot exclude the possibility that dose interval has an impact on lymph node-resident Tfh, which have previously been shown to be distinct from circulating Tfh following SARS-CoV-2 vaccination (44).

To further assess the quality of memory T-cell responses, we measured cytokines in the culture supernatants. After vaccination, IFN-γ, IL-2, TNF-α, granzyme B, IL-4, IL-5, and IL-13 were produced in response to Spike peptide stimulation, suggesting a polyfunctional response involving multiple T-cell subsets. Dose interval did not impact cytokine responses at any time point. Our data are limited by the analysis of cytokines in supernatants, rather than by intracellular cytokine staining (ICS), which would facilitate a more granular appraisal of cell type-specific cytokine responses. However, other studies employing ICS for the analysis of responses found conflicting results for IFN-γ (10, 11) and IL-2 (9, 10), which may be a consequence of subset-specific effects (10) and heterogeneity in T-cell dynamics (15). This heterogeneity poses challenges for identifying subtle differences in the quality of T-cell responses in small cross-sectional cohort studies.

The third vaccine dose boosted the proportion of AIM^+^ memory CD8^+^ T cells detected following SARS-CoV-2 Spike stimulation, but dose interval had no impact on the magnitude of the change resulting from the third dose, nor on memory CD4^+^ T-cell responses. Subsets of memory CD8^+^ or CD4^+^ T cells and T helper subsets among Spike-specific T cells were stable in pre- and post-boost samples. Similarly, the third dose had no effect on cytokine responses, irrespective of dose interval. These results are consistent with other data showing minimal impact of a third dose on long-term memory T-cell responses (45). Taken together, these data underscore the heterogeneity and stability of memory T-cell responses to mRNA-based COVID-19 vaccination, regardless of dose intervals. Alongside the observation that vaccine-primed memory T cells show wide recognition of variants (46, 47), these data reinforce the evidence that even as circulating antibodies decline, vaccine-primed cell-mediated memory responses offer sustained immune protection against severe SARS-CoV-2 outcomes in the case of infection.

Collectively, this study suggests that extending the interval between the first and second mRNA-based COVID-19 vaccine doses has a limited positive impact on the humoral response against SARS-CoV-2, resulting in increased durability of anti-Spike and anti-RBD binding antibody titers and improved neutralization of variants that are closely related to the vaccine immunogen. However, extending the dose interval did not improve antibody breadth sufficiently to confer protection against more divergent variants. A third (booster) dose further improves antibody magnitude and quality, irrespective of the primary dose interval. This suggests that the impact of dose interval is transient and is overridden by subsequent exposures that bring out more mature antibodies from B cells that have been continuing to mature. In contrast to humoral and memory B-cell responses, lengthening of the dose interval has minimal impact on antigen-specific memory T-cell responses, which demonstrate polyfunctionality and relative stability after two doses of vaccine and are not strongly affected by a third dose.

Taken together with previous data showing improved vaccine efficacy associated with extended dose intervals (14, 48), these findings support extending the primary dose interval of mRNA-based vaccines in specific situations with low vaccine supply or low burden of circulating virus. Indeed, in the absence of boosters, these immunological benefits may be sustained in the long term. However, our data suggest that these benefits of an extended dose interval are transient, with comparable immune responses observed between groups after the third dose. This suggests that shorter dose intervals, which provide maximal short-term protection in high viral prevalence settings, are not expected to have any long-term adverse consequences on vaccine-induced immunity. Although the majority of the population has now been exposed to SARS-CoV-2 through vaccination and/or infection, this has implications for optimizing childhood vaccination schedules and for the rollout of new mRNA vaccines for other infections.

## Data Availability

The original contributions presented in the study are included in the article/**Supplementary Material**. Further inquiries can be directed to the corresponding author.
