# Supplementary material for: Improved antibody breadth with an extended primary dose interval of COVID-19 vaccine is overcome by boosters
Source: Front Immunol. 2025 Mar 27;16:1529134. doi: 10.3389/fimmu.2025.1529134 (PMC11983636; doi:10.3389/fimmu.2025.1529134)
Supplement: Supplementary file 1 [file DataSheet1.docx]

**SUPPLEMENTARY MATERIALS**

| **Supplementary Table 1.** T cell AIM flow cytometry panel | | | |
| --- | --- | --- | --- |
| **Marker** | **Fluorochrome** | **Antibody clone** | **Source** |
| CD134 (OX40) | BB700 | ACT35 | BD Biosciences |
| CD137 (4-1BB) | BUV737 | 4B4-1 | BD Biosciences |
| CD69 | BUV805 | FN50 | BD Biosciences |
| CD3 | BUV563 | UCHT1 | BD Biosciences |
| CD4 | BB515 | RPA-T4 | BD Biosciences |
| CD154 (CD40L) | APC-Cy7 | TRAP-1 | BD Biosciences |
| CD14 | PECy5 | M5E2 | Biolegend |
| CD19 | PECy5 | HIB19 | BD Biosciences |
| CD8 | BUV395 | RPA-T8 | BD Biosciences |
| CD45RA | BV480 | HI100 | BD Biosciences |
| CD183 (CXCR3) | BV605 | G025H7 | Biolegend |
| CD194 (CCR4) | BV650 | 1G1 | BD Biosciences |
| CD196 (CCR6) | BV786 | 11A9 | BD Biosciences |
| CXCR5 | BB790-P | RF882 | BD Biosciences |
| CD197 (CCR7) | PECy7 | 3D12 | BD Biosciences |
| Zombie UV viability dye | -- | -- | Biolegend |


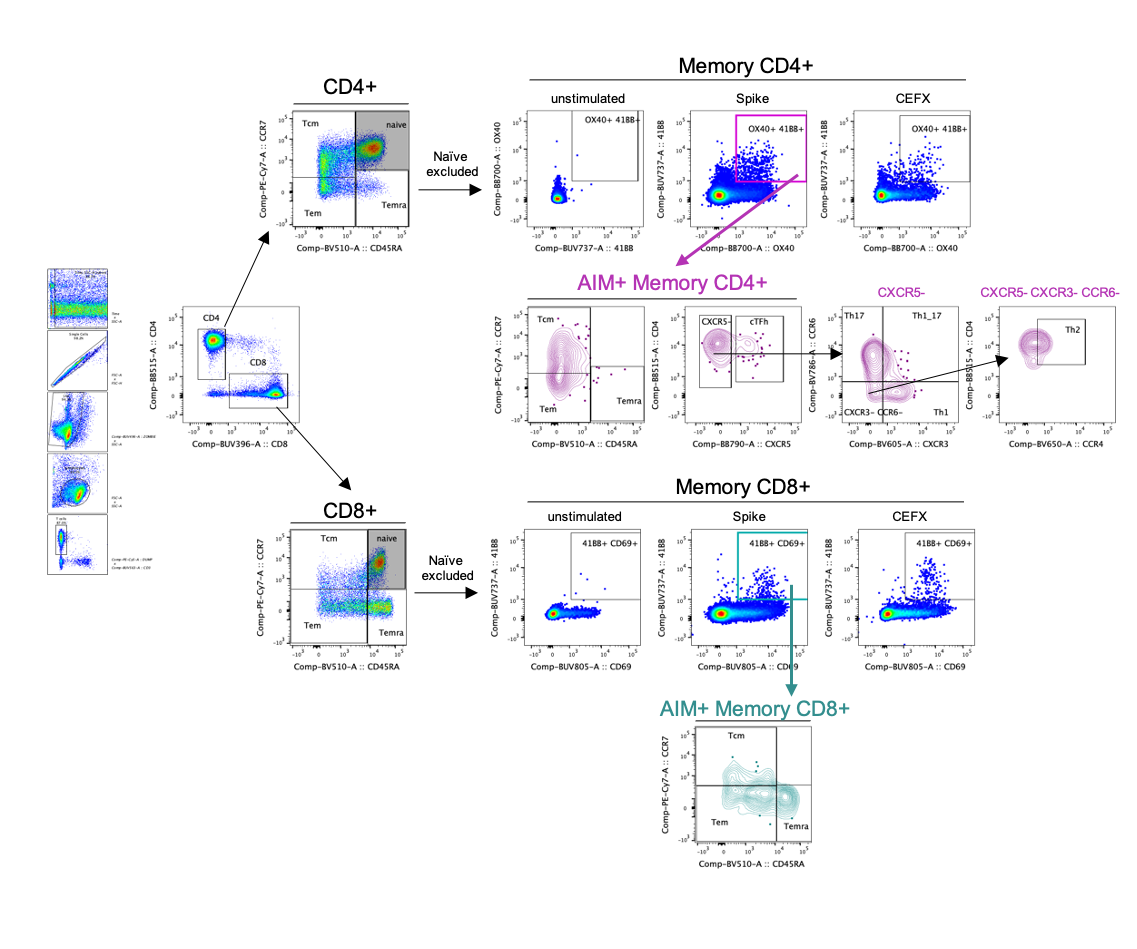
**Supplementary Figure 1.** Flow cytometry gating strategy for AIM assay.


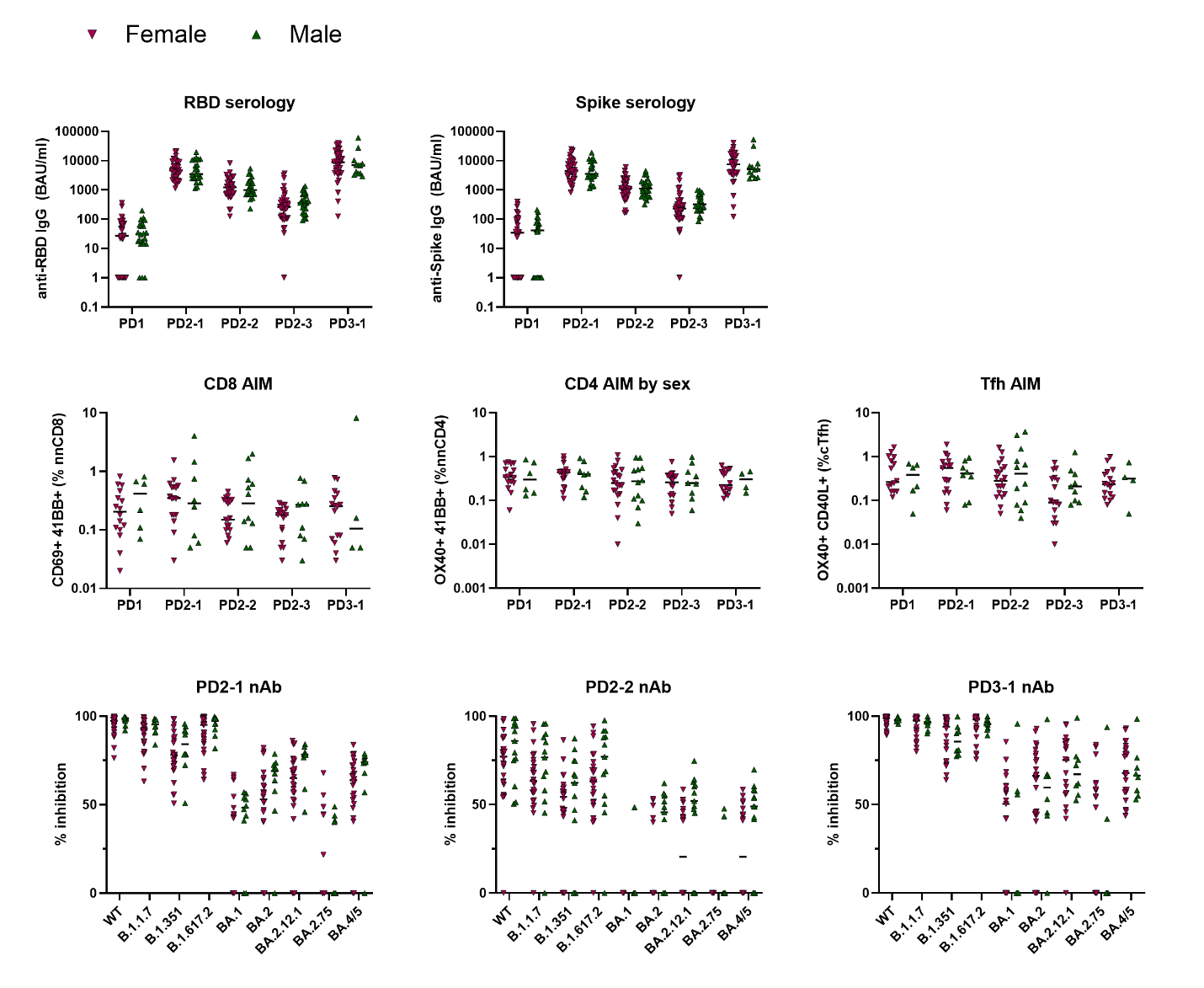


**Supplementary Figure 2**. Sex-based analysis of immune outcomes in COVID-19 Vaccine recipients. Each parameter was analyzed using Mann-Whitney tests with Holm-Sidak correction. Lines indicate median values.


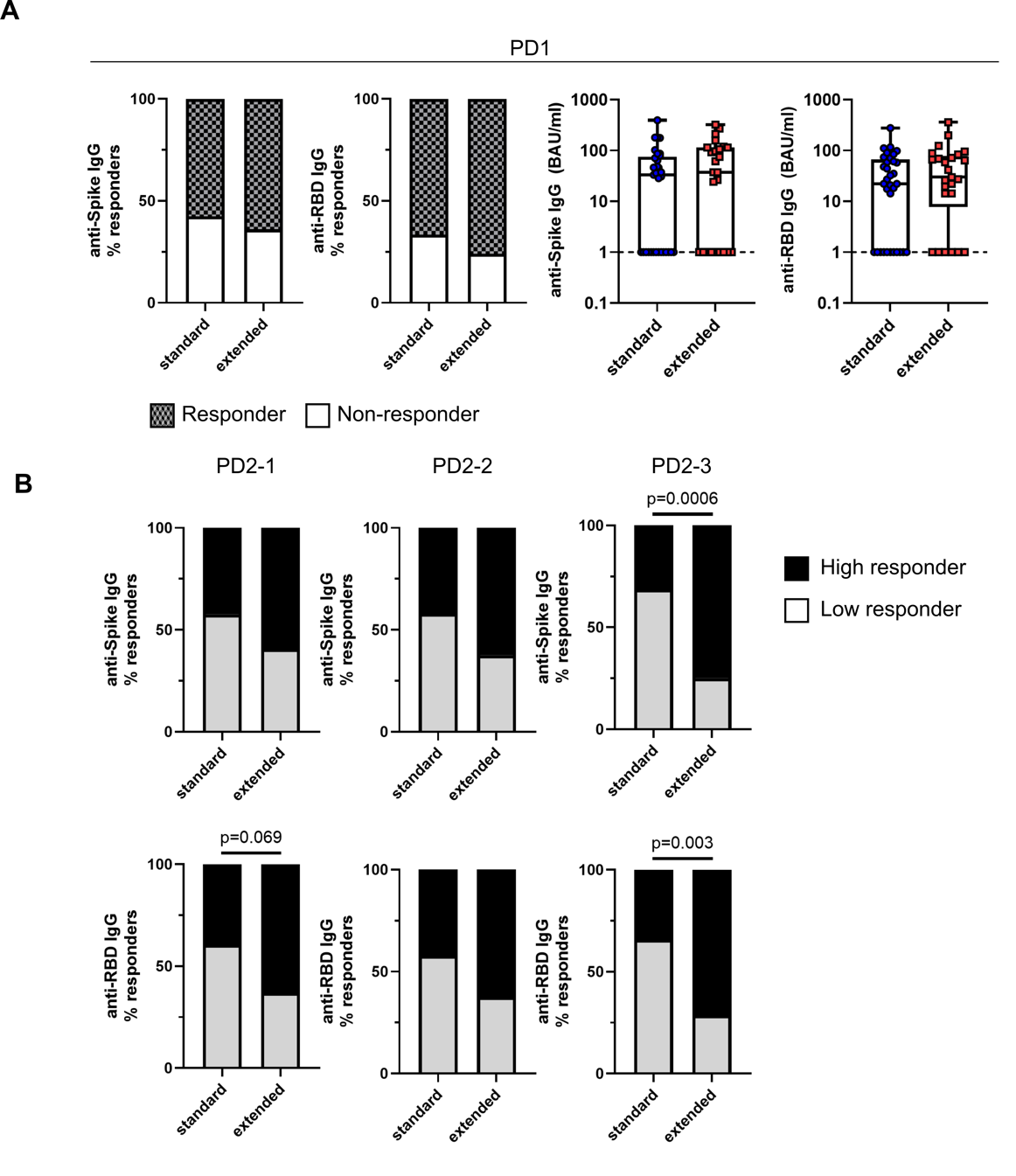


**Supplementary Figure 3.** Serological responses to vaccination. (A) Comparison of dose 1 serological responses between standard and extended interval groups. Left panels show proportion of participants who had detectable Spike- or RBD-specific IgG (responders, grey checked bar) vs. undetectable IgG (white bars) at PD1. Right panels show titers of Spike- and RBD-specific IgG at PD1. (B) Proportion of participants in the standard vs. extended interval groups who had Spike- or RBD-specific IgG titers at or above the median (high responders, black bars) or below the median (low responders, grey bars). Proportions compared between groups by Fisher’s exact test. Antibody titers compared between groups by Mann-Whitney U test.


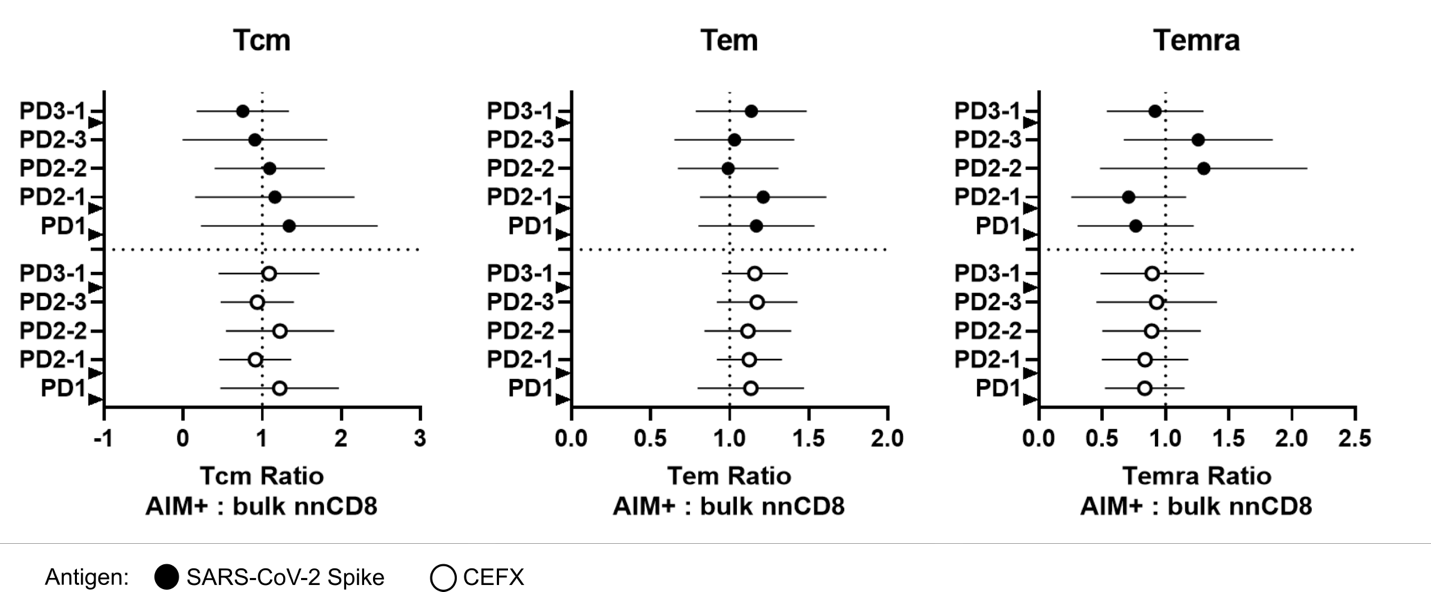


**Supplementary Figure 4**.Ratio of Spike-specific (closed circles) and CEFX-specific (open circles) CD8+ T cell subsets to proportion of subsets among bulk memory CD8+ T cells over time. A ratio of 1 indicates that the memory phenotype of antigen-specific cell population is proportionally representative of memory CD8+ T cells with other antigen specificities. Symbols indicate mean ratios. Bars represented standard deviation
